# Supplementary material for: Association between oral microbiome and seven types of cancers in East Asian population: a two-sample Mendelian randomization analysis
Source: Front Mol Biosci. 2023 Nov 21;10:1327893. doi: 10.3389/fmolb.2023.1327893 (PMC10702768; doi:10.3389/fmolb.2023.1327893)
Supplement: Supplementary file 1 [file DataSheet1.ZIP › Supplementary Materials/MR plots of saliva/saliva═╝/Prostate cancer/pheno.2938_to_prostate cancer_leave_one_out.pdf]

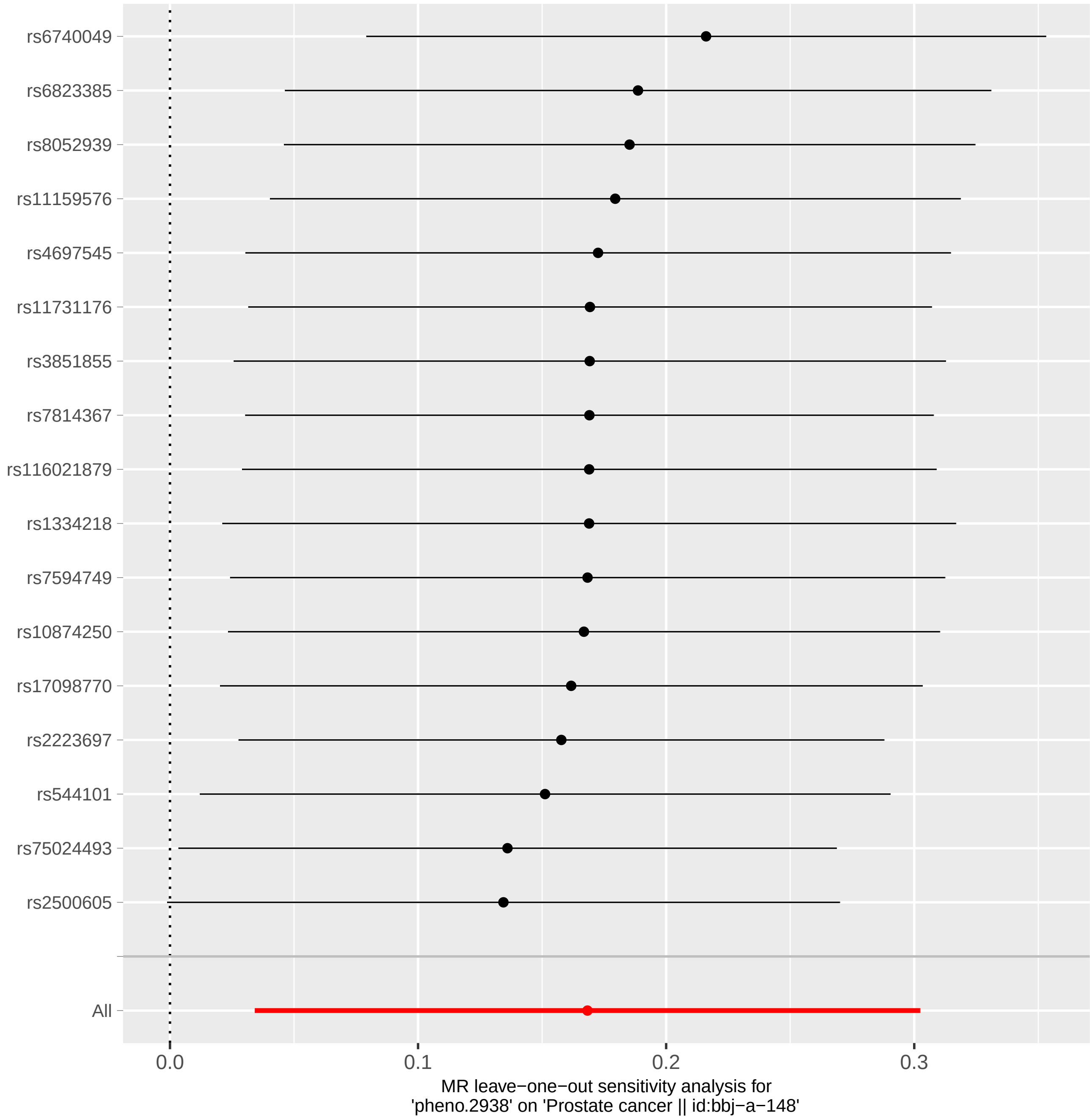

rs6740049
rs6823385
rs8052939
rs11159576
rs4697545
rs11731176
rs3851855
rs7814367
rs116021879
rs1334218
rs7594749
rs10874250
rs17098770
rs2223697
rs544101
rs75024493
rs2500605
All
0.0
0.1
0.2
0.3
MR leave−one−out sensitivity analysis for
'pheno.2938' on 'Prostate cancer || id:bbj−a−148'
